# Assessment of the Nutritional and Medicinal Potential of Tubers from Hairy Stork’s-Bill (*Erodium crassifolium* L ’Hér), a Wild Plant Species Inhabiting Arid Southeast Mediterranean Regions

**DOI:** 10.3390/plants9091069

**Published:** 2020-08-20

**Authors:** Shabtai Cohen, Hinanit Koltai, Gopinath Selvaraj, Moran Mazuz, Moran Segoli, Amnon Bustan, Ofer Guy

**Affiliations:** 1 Ramat Negev Desert Agro-Research Center (RN-DARC), Ramat Negev Works Ltd., D.N. Halutza 8551500, Israel; sab@inter.net.il (S.C.); moran.segoli@gmail.com (M.S.); amnonbustan@gmail.com (A.B.); 2 Department of Ornamental Horticulture and Biotechnology, Institute of Plant Sciences, Agricultural Research Organization, Rishon LeZion 7528809, Israel; hkoltai@volcani.agri.gov.il (H.K.); gopinath@volcani.agri.gov.il (G.S.); moranjacobi@gmail.com (M.M.)

**Keywords:** anti-inflammatory activity, antioxidants, catechin, domestication, *Erodium crassifolium*, underutilized species

## Abstract

Emerging needs for diversifying human diet and to explore novel therapeutic procedures have led to increasing attempts to retrieve traditional nourishments and recruit beneficial wild plant species. Species of the genus *Erodium* (Geraniaceae) harbor medicinal indications and substances known from folklore and scientific research. Hairy stork’s bill (*Erodium crassifolium* L’Hér), is a small hemicryptophyte that inhabits arid southeast Mediterranean regions. *E. crassifolium* is among the very few Geraniaceae species known to produce tubers. Traditional knowledge holds that the tubers are edible and used by Bedouin tribes. However, no scientific information was found regarding nutrition or medicinal properties of these tubers. The objectives of our project are to unravel potential nutritional and medicinal benefits of the tubers, conduct initial steps towards domestication and develop agricultural practices enhancing *E. crassifolium* tuber yield and quality. Tubers show high water content (90%), low caloric value (23 Kcal 100^−1^ g) and considerable contents of minerals and vitamins. In addition, the tubers contain significant amounts of catechins and epigallocatechin, polyphenolic compounds known for their antioxidative, anti-inflammatory and antiproliferative activities. Furthermore, in vitro experiments demonstrated significant anti-inflammatory effects on human cell cultures. *E. crassifolium* is highly responsive to environmental changes; fertigation (700 mm) increased tuber yield by 10-fold, compared to simulated wild conditions (50–200 mm). These results indicate a significant potential of *E. crassifolium* becoming a valuable crop species. Therefore, there is a need for continued efforts in domestication, including ecotype selection, breeding, development of suitable agricultural practices and further exploration of its medicinal benefits.

## 1. Introduction

Human diet and therapy have featured valuable plants gathered from the wild since ancient times. The agricultural civilization developed in the past 12,000 years has been founded on the domestication of many useful species [1]. However, in the modern era, global economic considerations have significantly changed agricultural approaches and scales, which brought about overexploiting of land resources, consistent diminishing of natural biodiversity and negative modifications of human diet and health [2,3]. Globalization and urbanization have accelerated the unification of the current human diet and have led to a further narrow hoard of available useful species [4]. The recent burst of noncommunicative diseases (NCD), including obesity, diabetes, cardiovascular disorders and cancer among populations of developing countries as well as lower socioeconomic classes of developed countries, is largely attributed to nutritional and health disorders derived from current human diets [5].

The emerging need for improving human diet has led to increasing interests in traditional nourishments, such as the Mediterranean diet [6] and in primordial therapeutic aids. These are usually based on diverse resources that change through locations, seasons and include numerous wild edible plant species. Attempts to retrieve old beneficial species and recruit them for new commercial use have a vast potential, considering that a very small portion of known plant species has ever been adequately studied for such purposes [7,8]. There is an increasing research activity focused on identifying and characterizing wild plant species with particular attributes to human diet and health [4,9,10,11,12,13,14,15]. Special attention is paid to edible plants as sources for essential mineral elements [16] and of antioxidative-active compounds [17]. In addition, there is a rising interest in plants encompassing anti-inflammatory activities [18].

Geraniaceae family includes seven genera and about 830 species distributed from temperate to tropic and arid climates. The largest genera are *Geranium* (430 species), *Pelargonium* (280 species) and *Erodium* (80 species). The family is known for the production of essential oils and ornamentals. Many Geraniaceae species are ascribed to have various medicinal values [19,20,21,22,23]. Additionally, Some of the species belonging to the genus *Erodium* have recognized medicinal indications from folklore and empirical data [24]. *Erodium* species are used to treat a variety of human ailments such as colds, coughs, diarrhea, hemorrhaging and are used to dress wounds [24,25,26,27,28].

**Figure 1:**
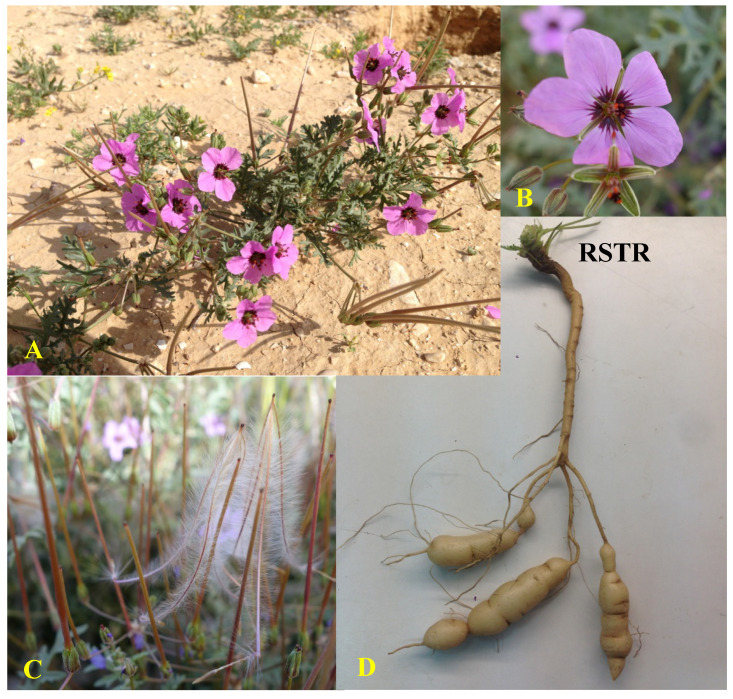
Hairy stork’s-bill (*E. crassifolium*). (**A**) Shrub with flowers and seed pods; (**B**) flower; (**C**) seeds with typical hairy feather-like awns; (**D**) root tubers, connected to the root-shoot transition region (RSTR), the perennial plant organ.

Hairy stork’s bill (HSB) (*Erodium crassifolium* L’Hér) is a Saharo-Arabian common perennial hemicryptophyte (i.e., buds are at or near the soil surface) that inhabits shrub–steppes of arid southeast Mediterranean regions. The species is distributed from northwest at Crete [29], through few Aegean Sea islands [30], the Libyan [31] and Egyptian [32,33] coasts, north Sinai Peninsula [32,34], Cyprus [35], the Negev Desert of Israel [36], until Edom mountains of Jordan and Saudi Arabian deserts, on southeast [37]. In Israel, HSB can be found in the Negev and Judean deserts, where the annual rainfall is 30–250 mm. The species is most abundant in the stony and arid loess soils and on slopes of limestone hills [36]. Vegetative phase in form of rosette (early season) and 30 cm stems and leaves (late season) starts with the first effective rains. Flowering time is February to May and the flowers are pink to purple with dark color base (Figure 1A,B) that attracts pollinating insects. The ripe ovary splits into five diaspores, each contains one seed covered by the ovary hairy wall with a sharply pointed tip (Figure 1C). The diaspores are carried away from the mother plant by strong winds and react to humidity changes by creating screw-like twists to penetrate the soil [36]. Among all Geraniaceae species, *E. crassifolium* is among the few known to produce tubers (Figure 1D). The tubers are formed on roots at depth of 5–20 cm and are typically small and spherical (1–2 cm in diameter). HSB tubers have a sweet taste and are best in late winter or early spring when they are whitish in color [36]. Traditional knowledge holds that the tubers are edible and Bedouin tribes are their primary consumers [33]. However, no information exists regarding nutritional value of these tubers. Furthermore, in spite of enduring claims associating some medicinal properties with HSB tubers, no supportive documented evidence have been found so far.

The objective of the present study was to evaluate, for the first time, the nutritive and medicinal potential of *E. crassifolium* for becoming a useful new crop species. In addition to a moderate nutritive potential, we demonstrate here significant in vitro anti-inflammatory capacities in the tubers, assigned to well-known bioactive compounds. Furthermore, HSB displayed impressive tuber productivity when exposed to agricultural conditions, indicating a promising potential for domestication.

## 2. Results and Discussion

The domestication of plant species is a complex iterating process, which includes selection according to subjective preferences [38], such as color, palatability and size. In the modern era, however, with the increasing awareness to the potential nutrition benefits or hazards of a given food product, an established list of nutrition facts has become an essential step in the initial evaluation of a candidate species. Here, for the first time, a comprehensive nutrition facts list is presented for HSB tubers (Table 1). The very high water content of HSB tubers makes them highly valuable for nomads in the hot dry desert, supporting the knowledge about their traditional use by Bedouin tribes [33]. Tubers’ caloric content is about half than in carrots, but they provide considerable amounts of essential minerals such as calcium, sulfur, magnesium and iron, as well as phosphorus and potassium. In addition, HSB tubers are a good source of vitamins A and C, harboring about a quarter and a third of their contents in carrot, respectively (Table 1).

Compared to carrots, the attractiveness of the HSB tuber in the fresh product market is quite moderate; it is crunchy, but inadequately sweet with no flavor. HSB tubers improve when served cooked, but further culinary efforts are required. As for any wild species, a long course of selection and breeding would be necessary to bring HSB tubers to a status of a common food produce.

**Figure 2:**
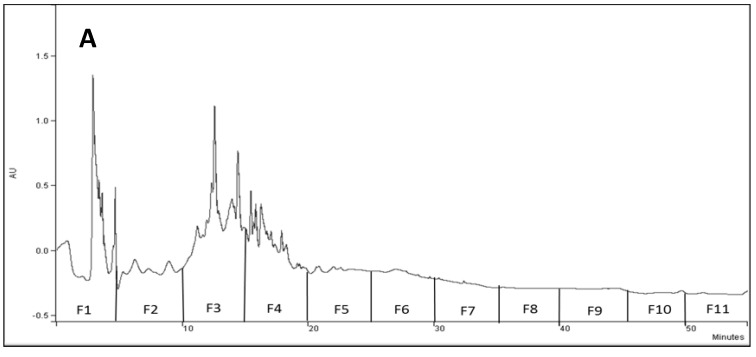

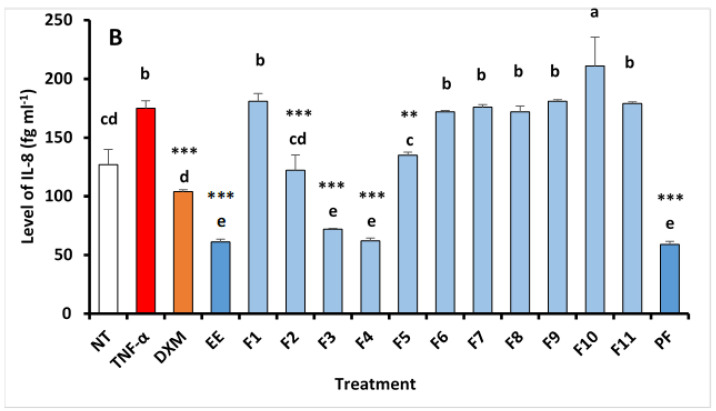
(**A**) HPLC profile of 70% ethanol extract (EE) of HSB tubers at 220 nm. Each fraction (F1–F11) was collected during 5 min out of the total 55 min of HPLC run; (**B**) levels of IL-8, an indicator of cell inflammatory status, in response to treatment with crude EE, EE fractions (F1–F11) and pooled fractions (PF) in in vitro trials using human cells. NT—non-treated control; TNF-α—an inflammation excitatory factor; DXM—dexamethasone (100 μM), positive control. Means of replicates were subjected to statistical analysis using Tukey–Kramer multiple comparison test. Different letters indicate significant differences between treatments; ***, ** and *, indicate *p* < 0.001, 0.01 and 0.05, respectively. Bars indicate standard error.

Enduring claims associating some medicinal properties with HSB tubers, so far with no supportive documented evidence, have encouraged our curiosity. Electromechanical analysis of the water-soluble extract from HSB tubers revealed significant reducing power, identifying at least six substances or groups of antioxidants (data not shown). Fractionation of the tubers’ ethanolic extract (EE) into 11 fractions (Figure 2A) and subsequent in vitro evaluations of possible anti-inflammatory capacities revealed significant activity in fractions F3 and F4, as well as in the original EE and the pooled fractions, PF (Figure 2B).

Biochemical analyses showed that fraction F4 comprised mainly of epigallocatechin, trans- and cis-catechin and gallic acid (Table 2), all of which are known for their robust bioactive capacities, including anti-inflammatory activity [40,41,42,43,44,45,46]. Interestingly, the bioactivity of fraction F4 was significantly greater than that of each compound alone at its corresponding concentration (data not shown), indicating synergic relationships in the natural extract. Furthermore, HSB tubers’ extract displayed significantly stronger anti-inflammatory capacity compared to extracts of green tea (*Camellia sinensis*) or turmeric (*Curcuma longa*) [47], well-known sources of antioxidative and bioactive compounds [40,48].

**Figure 3:**
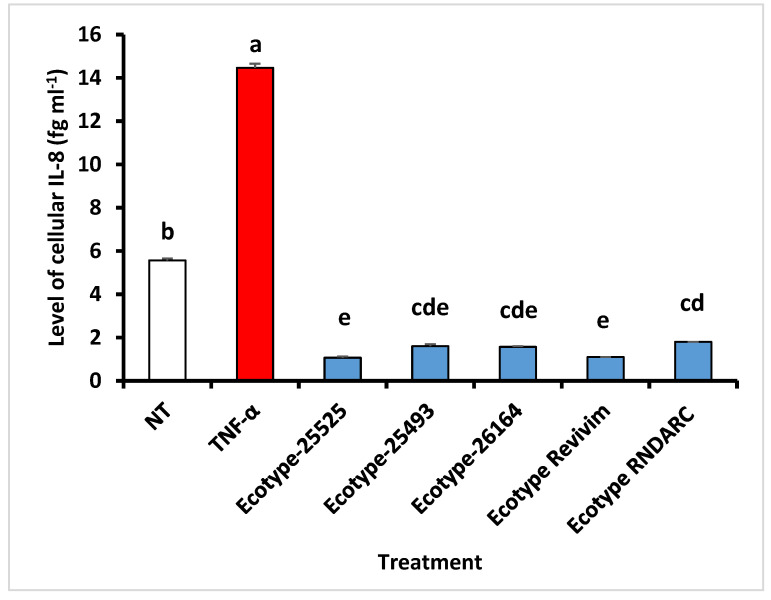
In vitro indications of the anti-inflammatory activity in the ethanolic extract from tubers of various *E. crassifolium* ecotypes, compared to untreated (NT) and stimulated (TNF-α) human cells. Bars indicate standard error. Different letters indicate significant differences between treatment at *p* < 0.05.

Screening several HSB ecotypes collected from diverse locations in Israeli deserts revealed that they all share a similar range of substantial anti-inflammatory capacity; however, there were considerable differences among the ecotypes (Figure 3), indicating that the natural diversity within the species may offer a promising potential for genetic enhancement of the tubers’ bioactive capacities. Accordingly, in the recent few years, seeds were collected from the wild across the Negev Desert and parts of the Rift Valley in order to broaden this potential.

Beyond selection and breeding, optimization of the growth conditions through irrigation, soil fertilization and weeding are founding principles of agriculture [38,49,50] and positive responses of a given wild plant species to these manipulations are prerequisite to successful domestication.

In the wild, HSB is a classic opportunist desert shrub, the proliferation of which largely depends on the current water availability during a given growth season. In arid conditions, the occurrence and intensity of late autumn rain events determine the rate of seed germination and of young seedlings survival. Later on, during winter and early spring, the extents of canopy development, reproductive phase and the duration of the growing season are governed by the intermittent desert precipitation regime [51].

**Figure 4:**
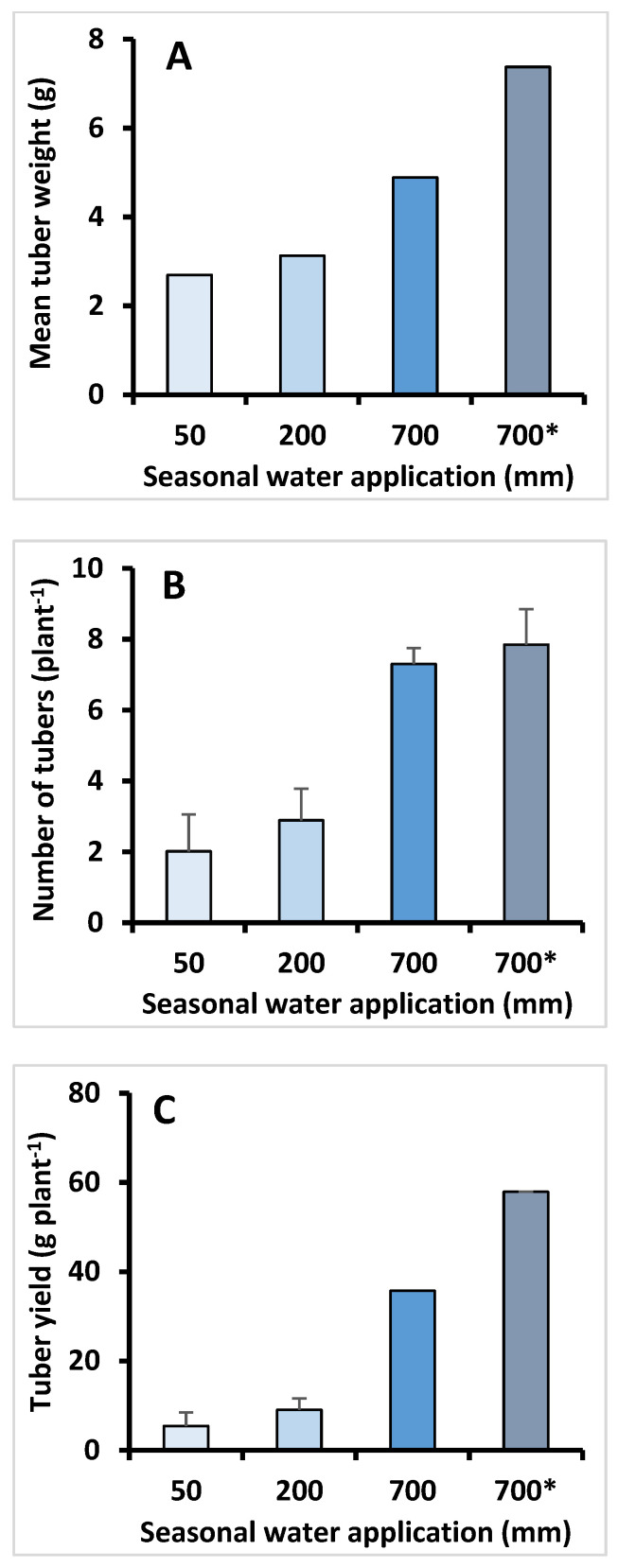
(**A**) Mean HSB tuber weight, (**B**) number of tubers and (**C**) mean tuber yield as affected by increased season water application at simulated natural precipitation patterns (50 and 200 mm) and at intensive agricultural environments (700 mm, drip irrigation and drip fertigation *); data were pooled from two different experiments conducted in the 2017–2018 season. Bars indicate standard deviation.

**Figure 5:**
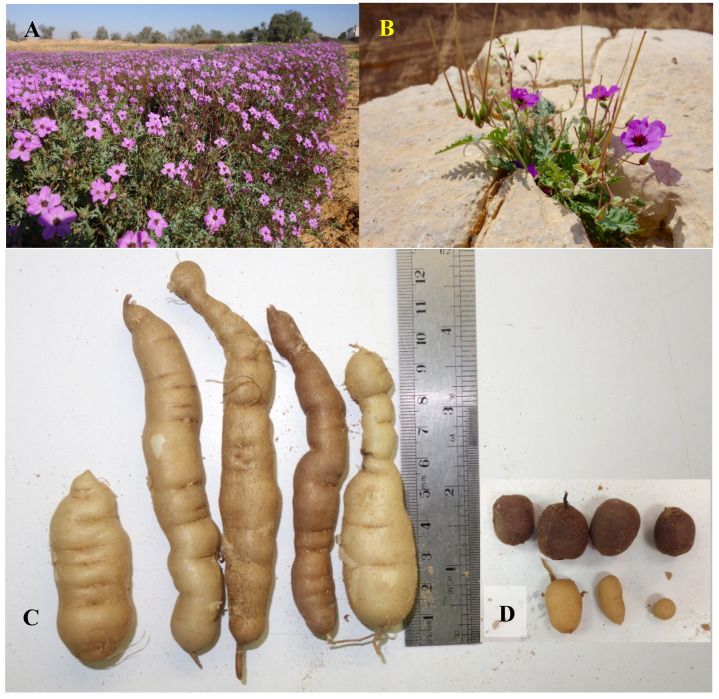
(**A**) Demonstration of an agricultural HSB cropping system at Ramat Negev with (**C**) representative tubers, compared to (**B**) the solitaire HSB phase in a wild niche, with (**D**) typical tubers’ size, form and age.

Simulation of various natural scenarios of the precipitation regime from November to May showed that in a relatively dry winter (50 mm), HSB exhibited low seed germination and seedlings survival rates. Conversely, many more plants persisted and grew in a rainy (200 mm) season (data not shown). Interestingly, the effects of water availability on tuber production per plant were very limited within the low precipitation range (Figure 4). However, tuber production and tuber weight increased significantly when water application increased to 700 mm, supplied consistently using drip irrigation, and no problems of germination or seedling persistence occurred. Moreover, plants produced significantly greater number (Figure 4B) of substantially larger tubers (Figure 4A). Adding fertilizer to the irrigated water had only a small influence on the number of tubers (Figure 4B), but tuber size increased by more than 50% (Figure 4A). Thus, application of a basic agricultural practice gave rise to a 10-fold increase in the mean tuber yield of an individual HSB plant (Figure 4C). Finally, the difference in plant and crop performance between wild and agricultural environments is unexpected (Figure 5); the greater germination and survival rates, fortified by enhanced tubers’ growth and development have brought about the current yield potential of cultivated HSB to about 15 Mg ha^−1^.

## 3. Materials and Methods

### 3.1. Ecosystem and Plant Material

The HSB project took place at Ramat Negev Desert Agro-Research Center (RN-DARC), Israel (30°58′ N 34°42′ E), 305 m above sea level. Soil texture varies across short distances from sandy dunes to sandy-loam Loess—and consequently differ in water retention and cation exchange capacity. The natural life cycle of HSB occurs in the rainy season, germinating from November and dispersing seeds up to May. Mean annual precipitation is about 82 mm, but the amount of rain can substantially vary among years and locations, as well as the distribution and intensity of rain events. During the growing season, daily average temperatures decline from 17.8 °C in November to about 10 °C in January and then steadily rise to 23 °C in May (Figure S1).

Most of the trials with HSB employed ecotype RNDARC (Accession No. 309198), which was collected near the trial site several years ago. Additional seed sources were ecotype Revivim (Accession No. 309199), the seeds of which were collected yearly near Kibbutz Revivim and three other accessions (ecotypes 25525, 25493 and 26164), kindly received from the Israeli Gene Bank, ARO.

### 3.2. Field Experiments

Two separate field experiments were conducted from November 2017 to June 2018. The first examined HSB growth performance in response to distinct precipitation regimes, whereas the second experiment evaluated it under two different agricultural practices.

#### 3.2.1. Simulated Precipitation Regimes

Treatments represent four principal scenarios of rainy seasons in the Negev Desert of Israel, differing in the intensity and frequency of rain episodes, as follows: A. low and concentrated (50 mm, comprised of two events); (B) low and scattered (50 mm, spread in ten events); (C) high and concentrated (200 mm, in four events); and, D. high and scattered (200 mm, in ten events) precipitation. Seeds were sown in loess soil at a density of eight seeds m^−2^, in 6 m^2^ plots. The experiment was organized in a random block design with four replicates. Irrigation was executed using computer-controlled sprinklers, and no fertilizer or soil amendments were used. The amount of water was monitored from germination and included rain. Plants were counted weekly. At harvest in June, tuber number and weight were determined per each surviving plant.

#### 3.2.2. Agricultural Practice

Seeds were sown at late November 2017 in an open field on sandy soil beds. Water was supplied using two drip lines per bed (40 cm apart), with five 1.6 L h^−1^ emitters per 1 m of drip line. Two seeds were sown on either side of each emitter, resulting in a density of 16 plants m^−2^. Fresh water (0.7 dS m^−1^) was supplied at 6 mm day^−1^ until germination, which occurred about 2 weeks after sowing. After germination, irrigation was reduced to 4 mm day^−1^ to accomplish 700 mm until late May. An unfertilized control was tested against a fertigated treatment, which was applied using a liquid fertilizer (Shefer 4:2:6, 35% ammonium and 65% nitrate, Israel Chemicals, Ltd.) at 1.5 L m^−3^. The experimental design was of random blocks with four replicates (plot size was 2.4 m^2^). Bloom began in April and continued until June. Tubers began forming at a very early stage of plant growth and continued to form and develop throughout the season. Tuber harvest took place in June, toward seeds ripening. At harvest, tuber yield was determined; tubers were cleaned and stored at −20 °C until further examinations.

### 3.3. Evaluation of Tubers Nutrition Facts

To assess HSB tuber nutrition facts (Table 1), samples (2 kg each) were sent to BactoChem, Ltd., Ness Ziona, Israel, an officially certified laboratory, tightly committed to AOAC protocols.

### 3.4. Ethanolic Extract (EE)

HSB tubers were removed from cold storage (−20 °C) and frozen in liquid nitrogen. The frozen tubers were homogenized using an electrical blender and weighed. For each 1 g of fresh material, 4 mL of 70% ethanol were added immediately to the crushed tubers and incubated overnight at 28 °C with shaking at 180 rpm, after which the samples were centrifuged in 50-mL tubes for 5 min at 2500 rpm in an Eppendorf 5810R centrifuge with a 26 cm rotor (1820 RCF). The supernatant was transferred to new tubes and the solvent was evaporated in vacuo overnight. The remaining water content was lyophilized to powder and stored at −20 °C. From each gram of tubers, approx. 60 mg of lyophilized extract was obtained.

Just before further analysis, the lyophilized material was weighed and dissolved in 100 μL of 70% ethanol and then 900 μL of double distilled water (DDW), to obtain a 60 mg mL^−1^ sample, which was filtered through a 0.45-μm membrane.

### 3.5. High-Performance Liquid Chromatography (HPLC) Analysis

The filtered EE sample was separated using an Ultimate 3000 HPLC system coupled with a WPS-3000(T) Autosampler, HPG-3400 pump and DAD-300 detector. The separation was performed using a Purospher RP-18 endcapped column (250 mm × 4.6 mm I.D.; Merck KGaA, Darmstadt, Germany) with a guard column (4 mm × 4 mm I.D.). Solvent gradients were formed by varying the proportion of solvent A [water with 0.1% acetic acid (*v/v*)] to solvent B (methanol) with a flow rate of 1.0 mL min^−1^. Solvent B was maintained initially at 10% for 5 min and then increased to 100% in 25 min. This 100% of Solvent B was maintained for 10 min, then decreased to 10% in 10 min and equilibrated for 5 min (total run time 55 min). The compound peaks were detected at three different wavelengths: 220, 240 and 280 nm. The same program was used to obtain fractions in bulk using a preparative HPLC (11250 Infinity, Agilent Technologies) using reversed-phase C18 column (Kinetex 5u EVO C18-100A—250 × 21.2 mm). After collection, the fractions were lyophilized to powder. These lyophilized fractions were resuspended in 7% ethanol and checked for their effect on IL-8 levels, as described below. Further analyses were carried out to correlate the activity and peak profile for detecting the active compound peak(s).

### 3.6. GC–MS Analysis

Prior to GC–MS analysis, samples underwent derivatization; 200 µL of N,O-bis(trimethylsilyl)trifluoroacetamide (BSTFA, Sigma-Aldrich, T-6381, USA) containing 1% of trimethylchlorosilane (TMCS) was added to each completely dried extract and heated to 70 °C for 20 min.

GC-MS analyses were carried out using a HP7890 gas chromatograph coupled to a HP6973 mass spectrometer (electron multiplier potential 2 KV, filament current 0.35 mA, electron energy 70 eV and the spectra were recorded over the range *m/z* 45 to 1000). An Agilent 7683 autosampler was used for sample introduction. Helium was used as a carrier gas at a constant flow of 1.1 mL s^–1^. One μL of each sample was injected into the GC–MS using a 1:10 split ratio injection mode. An isothermal hold at 50 °C was kept for 2 min, followed by a heating gradient of 6 °C min^−1^ to 300 °C, with the final temperature held for 4 min. A 30 m, 0.25 mm ID 5% cross-linked phenylmethyl siloxane capillary column (HP-5MS) with a 0.25-μm film thickness was used for the separation, and the injection port temperature was 220 °C. The MS interface temperature was 280 °C. Peak assignments were carried out with the aid of library spectra (NIST 14.0) and compared with published data and MS data obtained from the injection of standards [(-)-Epigallocatechin, 08,108; catechin, *U-*49,040; gallic acid, 91,215] purchased from Sigma-Aldrich, Switzerland or USA.

### 3.7. Human Cell Culture

HaCaT is a spontaneously transformed aneuploid immortal keratinocyte cell line from adult human skin [52], widely used in scientific research [53]. HaCaT (ATCC-HB-241) normal skin cells were grown at 37 °C in a humidified 5% CO_2_–95% air atmosphere. Cells were maintained in Dulbecco’s modified Eagle’s medium (DMEM, Biologic Industries, 01-055-1A, Beit-Haemek, Israel) with 10% fetal bovine serum (FBS, Biological Industries, 04-007-1A, Israel) and penicillin (100 units mL^−1^)—streptomycin (100 μg mL^−1^) solution (Biologic Industries, 03-031-1B, Beit-Haemek, Israel).

Determination of Interleukin 8 (IL-8) levels in HaCaT cells

HaCaT cells were seeded into 24-well plates at 50,000 cells per well in triplicate in 500μL of media and then incubated for 24 h at 37 °C in a humidified 5% CO2–95% air atmosphere. After incubation, cell excitation was performed with recombinant human tumor necrosis factor-α (TNF-α, PeproTech, 300-01A, Cranbury, NJ, USA). Cultures in each well were treated with a final concentration of 50 ng mL^−1^ of TNFα and 50 µL plant extract. Three different controls were included in all experiments: (a) untreated cells, with neither TNF-α nor plant extracts, (b) cells treated with TNF-α alone, (c) cells treated with TNF-α and the solvent (7% ethanol). The supernatant was taken, and the level of IL-8 was measured 16 h posttreatment using the commercial Human CXCL8/IL-8 DuoSet ELISA kit (R&D Systems, DY208-05, McKinley Place MN, USA) according to the manufacturer’s protocol. IL-8 is a common biomarker for inflammatory skin diseases [54,55]. The induction of detectable IL-8 levels requires a 16 h exposure to TNF-α [56]. Dexamethasone (Sigma-Aldrich, D4902, St. Louis, MO, USA) was used as a positive control [57,58].

## 4. Conclusions

Wild plant species may hold immense potential resources of nutritional value and therapeutic substances. In the present study, the tubers of *E. crassifolium*, an ignored desert shrub, were shown to harbor significant nutritional values and anti-inflammatory capacities. The catechins found in the tubers’ ethanolic extract have well-established remedial effects in serious human ailments. Furthermore, indications exist suggesting that HSB tubers have a greater medicinal potential. While a demonstrated bioactive capacity is a prerequisite, high productivity is essential in realizing the potential of an underutilized species. The dramatic increase in HSB tubers yield in response to fertigation paves the way for this plant to become a potential industrial crop.

## Figures and Tables

**Table 1:** Nutritional profile of hairy stork’s bill (HSB) tubers compared to that of carrots (adopted from USDA, 2020 [39]).

Nutritional Profile	Measure Units /100 g	HSB Tubers	Carrot
Water	g	90.6	88.3
Ash		0.8	0.9
Caloric value	Kcal	23	41
Carbohydrates	g	7.9	9.6
Sugars		4.3	6
Polysaccharides		3.0	2.8
Lipids		0.1	0.24
Protein		0.6	0.93
Potassium	mg	223.4	320
Calcium		74.3	33
Phosphorus		28.0	35
Sulfur		27.4	
Magnesium		20.0	12
Sodium		18.9	69
Iron		2.2	0.3
Zinc		0.4	0.24
Vitamin A	μg	<200	835
Vitamin C (ascorbic acid)	mg	2.03	5.9
A-tocopherol (vitamin E)	IU	<1	

**Table 2:** Compounds identified by GC-MS from fraction 4 (F4) of 70% ethanol extract of *E. crassifolium* tubers. RT—retention time.

Compound	RT (min)	Percentage (%) from Total Amount
Mannofuranose	28.024	17.2
α-D-xylopyranose	29.047	2.7
Gallic acid	29.766	5.7
Palmitic acid	30.853	6.4
Stearic acid	33.831	2.0
Trans-catechin	42.166	11.7
Cis-catechin	42.435	12.8
Epigallocatechin	43.007	41.5

## Supplementary Materials

The following are available online at https://www.mdpi.com/2223-7747/9/9/1069/s1, Figure S1: Mean monthly maximum, average, and minimum temperatures at RNDARC.
