# Supplementary material for: Assessment of the Nutritional and Medicinal Potential of Tubers from Hairy Stork’s-Bill (Erodium crassifolium L ’Hér), a Wild Plant Species Inhabiting Arid Southeast Mediterranean Regions
Source: Plants (Basel). 2020 Aug 20;9(9):1069. doi: 10.3390/plants9091069 (PMC7570100; doi:10.3390/plants9091069)
Supplement: Supplementary file 1 [file plants-09-01069-s001.pdf]

Supplementary

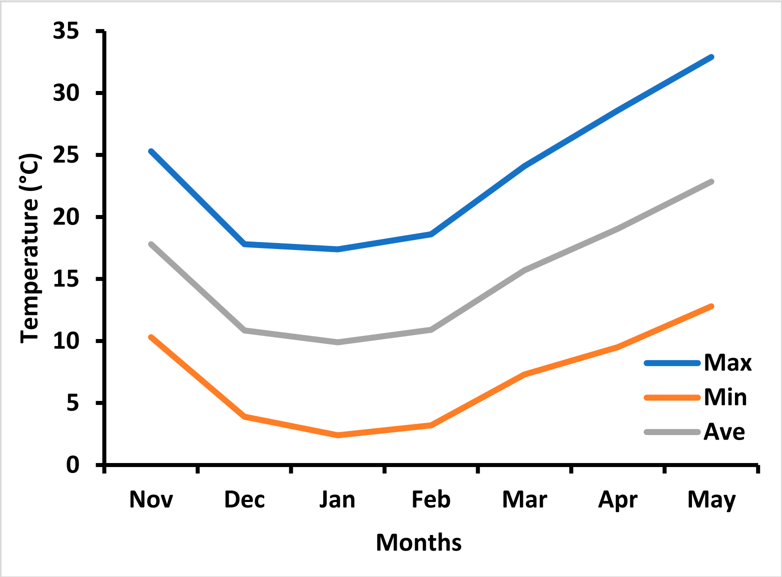

Figure S1. Mean monthly maximum, average, and minimum temperatures at RNDARC. Data are means of the recent 10 years (2010-2019).
